# On Chemical Bonding in *ht*-Ga_3_Rh and Its Effect on Structural Organization and Thermoelectric Behavior

**DOI:** 10.1021/acs.inorgchem.4c01280

**Published:** 2024-06-14

**Authors:** Raúl Cardoso-Gil, Mitja Krnel, Frank R. Wagner, Yuri Grin

**Affiliations:** Max-Planck-Institut für Chemische Physik fester Stoffe, Nöthnitzer Str. 40, 01187 Dresden, Germany

## Abstract

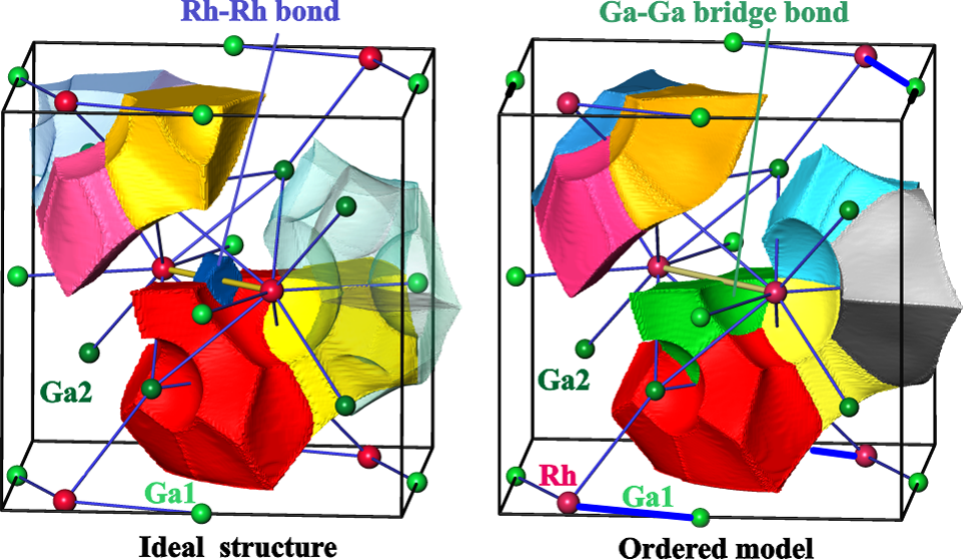

In the course of systematic studies of intermetallic compounds Ga_3_TM (TM—transition metal), the compound Ga_3_Rh is synthesized by direct reaction of the elements at 700 °C. The material obtained is characterized as a high-temperature modification of Ga_3_Rh. Powder and single-crystal X-ray diffraction analyses reveal tetragonal symmetry (space group *P*4_2_/*mnm*, No. 146) with *a* = 6.4808(2) Å and *c* = 6.5267(2) Å. Large values and strong anisotropy of the atomic displacement parameters of Ga atoms indicate essential disorder in the crystal structure. A split-position technique is applied to describe the real crystal structure of *ht*-Ga_3_Rh. Bonding analysis in *ht*-Ga_3_Rh performed on ordered models with the space groups *P*1̅, *P*4_2_*nm*, and *P*4_2_2_1_2 shows, besides the omnipresent heteroatomic Ga–Rh bonds in the rhombic prisms _∞_^3^[Ga_8/2_Rh_2_], the formation of homoatomic Ga–Ga bonds bridging the Rh–Rh contacts and the absence of significant Rh–Rh bonding. These features are essential reasons for the experimentally observed disorder in the lattice. In agreement with the calculated electronic density of states, *ht*-Ga_3_Rh shows temperature-dependent electrical resistivity of a “bad metal”. The very low lattice thermal conductivity of less than 0.5 W m^–1^ K^–1^ at 300 K, being lower than those for most other Ga_3_TM compounds, correlates with the enhanced bonding complexity.

## Abstract 2

Strong anisotropy of the ADPs of Ga atoms indicates essential disorder in the crystal structure of *ht*-Ga_3_Rh. Quantum chemical bonding analysis shows besides the omnipresent heteroatomic Ga−Rh interactions in the main building unit—rhombic prisms —the formation of homoatomic Ga−Ga bonds bridging the Rh−Rh contacts and the absence of Rh−Rh bonding. These features are essential reasons for the experimentally observed disorder in the lattice and low thermal conductivity.

## Introduction

The crystal structures of intermetallic compounds exhibit a rich variety of atomic arrangements generated by complex atomic interactions, which establish the basis for interpretation of the corresponding physical properties. Accordingly, in recent years, emphasis has been placed on the systematic study of both crystal and electronic structures of Ga_3_TM intermetallic compounds with transition metals (TM) from groups 8 and 9 of the Periodic Table, aiming at the improvement of their potential thermoelectric properties and the better understanding of the relation between chemical bonding and properties of these compounds.^1−4^ The intermetallic compounds with the composition Ga_3_TM (TM = Fe, Ru, and Os; Co, Rh, and Ir) were first reported with crystal structures isotypic to In_3_Ir (space group *P*4̅*n*2).^5,6^ Later, it was shown that the adequate symmetry of the structural arrangement in In_3_Ir is described by the space group *P*4_2_/*mnm* and suggested to assign the structure type Ga_3_Fe.^7^ Nevertheless, according to the structure type assignment rules the structure type notation in the databases is still IrIn_3_.^8^ Noteworthily, the latter corresponds to the tetragonal *ht*-IrIn_3_ modification since an orthorhombic *lt*-IrIn_3_ modification also occurs.^9,10^ A special feature in this series of intermetallic compounds is that Ga_3_TM (TM = Fe, Ru, and Os) compounds are—rather unexpectedly—narrow band gap semiconductors,^11^ whereas Ga_3_TM (TM = Co, Rh, and Ir) show metallic character.^11,12^ However, according to theoretical calculations,^11,13^ Ga_3_Ir should be also a semiconductor. This feature was experimentally confirmed by electrical resistivity measurements on single-phase polycrystalline material of *ht*-Ga_3_Ir (space group *P*4_2_/*mnm*, stable between 799 and 964 °C).^14^ It exhibits lattice thermal conductivity lower than other Ga_3_TM materials.^4,14^ Furthermore, the low-temperature modification *lt*-Ga_3_Ir with crystal structure isotypic to Al_3_Ni (or Fe_3_C according to ref (8)) (space group *Pnma*) is found to be stable below 530 °C.^15^ Detailed analysis of chemical bonding in Ga_3_Fe reveals the transition metal as the negatively charged component.^16^ This is also confirmed in the present study (cf. Results and Discussion). Thus, the chemical composition of these compounds is Ga_3_TM, which is used hereafter in this contribution.

An outline of the Ga-rich side of the phase diagram was proposed, based on the results of differential thermal analysis (DTA^17^). However, a complete phase diagram of the Ga–Rh system is still missing. Several binary compounds in the Ga–Rh system have already been identified and described in the literature. On the Ga-rich side, the compounds Ga_16_Rh_3_,^18^ Ga_21_Rh_4_,^18^ and Ga_9_Rh_2_^19,20^ have been reported. Besides crystal structure reports^5,6^ and the study on the enthalpy of formation,^17^ no further information has been found in the literature on Ga_3_Rh. Therefore, to complete our systematic study on the structure and properties of Ga_3_TM intermetallic compounds, we resynthesized *ht*-Ga_3_Rh, revisited the crystal structure, performed bonding analysis, and characterized thermoelectric properties.

## Experimental Section

### Preparation

Ga_3_Rh samples were prepared from elemental gallium (99.9999%, drops, Alfa Aesar) and rhodium (99.9%, pieces, Alfa Aesar). For the reaction, the starting materials were placed in quartz glass ampules sealed under a vacuum (10^–6^ mbar). The ampule was placed hanging in a vertical furnace for heat treatment consisting of a heating step of 12 h up to 700 °C, followed by a dwell of 168 h at this temperature, and the final quick quenching of the ampule in ice/water. No reaction of educts and products with quartz was observed. Additionally, the samples were ground in a mortar, cold-pressed to a pellet (Ø = 8 mm), sealed in a quartz glass ampule, and annealed for a second time with the same temperature–time profile as described before.

### Characterization

Pieces of annealed samples were embedded in conductive resin (PolyFast, Struers, Denmark), ground, and polished (using microsized diamond powders) for metallographic analysis. Sample homogeneity and chemical composition were analyzed on a scanning electron microscope (CAMECA SX100 Electron Microprobe) by standard-based wavelength dispersive X-ray spectroscopy (WDXS). The spectral lines Rh Lα and Ga Kα were employed using elemental Rh and GaP as standards for rhodium and gallium, respectively.

The thermal behavior of the Ga_3_Rh samples was evaluated on a DSC 404C differential scanning calorimeter (DSC, NETZSCH GmbH & Co.) in the temperature range from room temperature (RT) to 1000 °C (Al_2_O_3_ corundum crucibles, sample mass ∼40 mg, and heating rate of 10 K min^–1^).

Powder X-ray diffraction (PXRD) analysis was used in different steps of the experiments. For phase identification and lattice parameter determination, PXRD data were collected on an Image Plate Guinier Camera Huber G670 (Cu Kα_1_ radiation, λ = 1.54056 Å, 5° ≤ 2θ ≤ 100°; Δθ = 0.005°). The program packages WinXPOW^21^ and WinCSD^22^ were used for qualitative phase analysis and all crystallographic calculations, respectively. The lattice parameters were determined by least-squares refinement on 65 reflection positions in the range of 15° < 2θ < 100°, using LaB_6_ (*a* = 4.1569 Å) as internal standard.

High-temperature powder X-ray diffraction experiments were performed with synchrotron radiation (λ = 0.40005 Å) at the ID22 high-resolution powder diffraction beamline at the ESRF, Grenoble. For these experiments, samples are ground and a sieved fraction between 25 and 32 μm particle size is filled into a 0.2 mm diameter quartz capillary for data collection. A temperature-controlled hot-air blower is used for sample heating from room temperature up to 800 °C.

Selected crystals with nonregular shapes were fixed with glue at the top of a glass needle each. Single-crystal X-ray diffraction intensity data collection at room temperature was performed on a Rigaku AFC7 diffractometer system (Mo Kα radiation, λ = 0.71073 Å, graphite monochromator).

A regular bar-shaped specimen (*l* = 3.98 mm, *w* = 1.95 mm, and *h* = 1.36 mm) was cut from the pelletized and annealed material for the simultaneous measurement of thermoelectric properties (electrical resistivity ρ, Seebeck coefficient *S*, and thermal conductivity κ) at low temperature (5 K ≤ *T* ≤ 350 K), using the thermal transport option (TTO) of the Physical Property Measurement System (PPMS, Quantum Design, San Diego).

### Quantum Chemical Calculations

Electronic structure calculations and chemical bonding analysis were carried out employing the all-electron, local orbital full-potential method (FPLO) within the local density approximation^23^ and the Pedrew–Wang parametrization^24^ (scalar relativistic calculation, standard basis set, 12 × 12 × 12 *k* points). The experimental crystallographic information was used. Solely for the sake of bonding studies on *ht*-Ga_3_Rh, four models were developed to reflect possible atomic arrangements in the split model at the highest possible symmetry (Table S1).

For the analysis of chemical bonding in position space, the electron density (ED) and the electron localizability indicator (ELI-D) were calculated with a module implemented in the FPLO program package.^25^ The computed distributions of ED and ELI-D were analyzed with the program DGrid.^26^ For this purpose, the electron density was integrated within atomic basins, i.e., spatial regions confined by zero-flux surfaces in the gradient field of ED and ELI-D. This technique represents the procedure proposed in the Quantum Theory of Atoms in Molecules (QTAIM^27^) and provides effective electron populations for the QTAIM atoms and ELI-D bond basins. Further information about the bonding between atoms is obtained from the electron-localizability approach, a combined analysis of ED and ELI-D.^28^

## Results and Discussion

### Preparation

**Figure 1:**
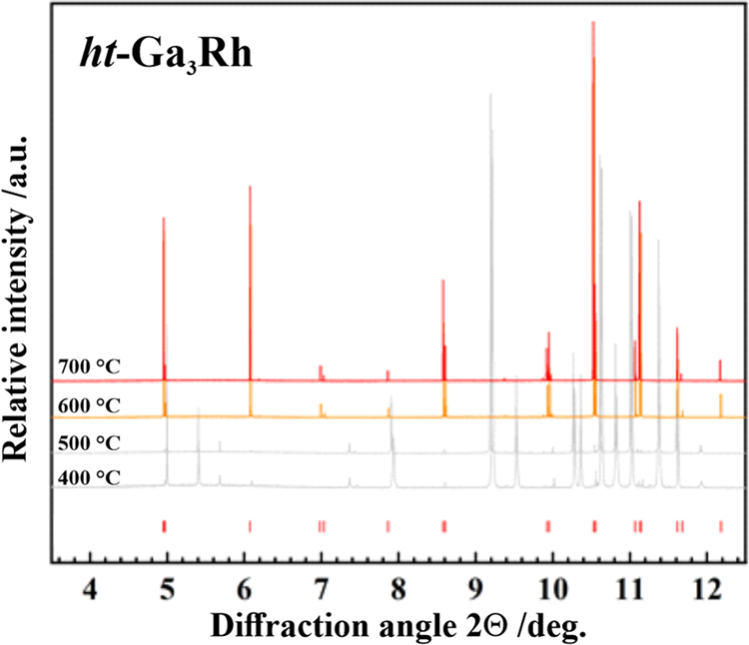
Powder X-ray diffraction patterns (synchrotron radiation, λ = 0.400052 Å, temperature range 400 °C ≤ *T* ≤ 700 °C) of the Ga_3_Rh sample, prepared by quenching after annealing at 700 °C. The patterns at 400 and 500 °C show reflections of the *lt-*Ga_3_Rh phase.^33^ The pattern at 600 °C already exhibits reflections of the *ht* phase with the tetragonal IrIn_3_ type (space group *P*4_2_/*mnm*, red bars at the bottom).

The existence of gallium-rich compounds with Ga content higher than 75 atom % in the Ga–Rh system prevents the use of the self-flux method, i.e., crystallization from the two-phase region under liquidus,^29–32^ for the synthesis of Ga_3_Rh. Thus, the direct heat treatment of stoichiometric amounts of elemental Ga and Rh was chosen for the preparation of this material. To determine the reaction temperature, a preliminary DSC analysis was performed on mixtures of elemental Rh and Ga in a molar ratio of 1:3. First attempts resulted in rather unclear DSC profiles and complex PXRD patterns of the products, suggesting a multiphase system. These outcomes resembled the behavior of Ga_3_Ir, where two modifications—high- and low-temperature—occur.^14,15^ Therefore, aiming to determine the stability range of possible modifications in the case of Ga_3_Rh, a series of samples was annealed and quenched from specific temperatures selected from the DSC data. One of these samples, quenched from 700 °C and containing >98 vol % of Ga_3_Rh with the tetragonal crystal structure of the IrIn_3_-type was selected to perform PXRD at higher temperatures using synchrotron radiation (for experimental details see above). The X-ray diffraction patterns in the temperature range from 400 to 700 °C (Figure 1) show first the transformation from the *ht*-Ga_3_Rh modification, metastable at room conditions, to the *lt*-Ga_3_Rh one (400 and 500 °C, cf. DSC results below). This low-temperature modification of Ga_3_Rh was characterized and will be reported separately.^33^ The *ht*-Ga_3_Rh phase reappears in the PXRD pattern at 600 and 700 °C. The latter PXRD pattern was indexed in the tetragonal symmetry (space group *P*4_2_/*mnm*; cf. red bars at the bottom of Figure 1).

**Figure 2:**
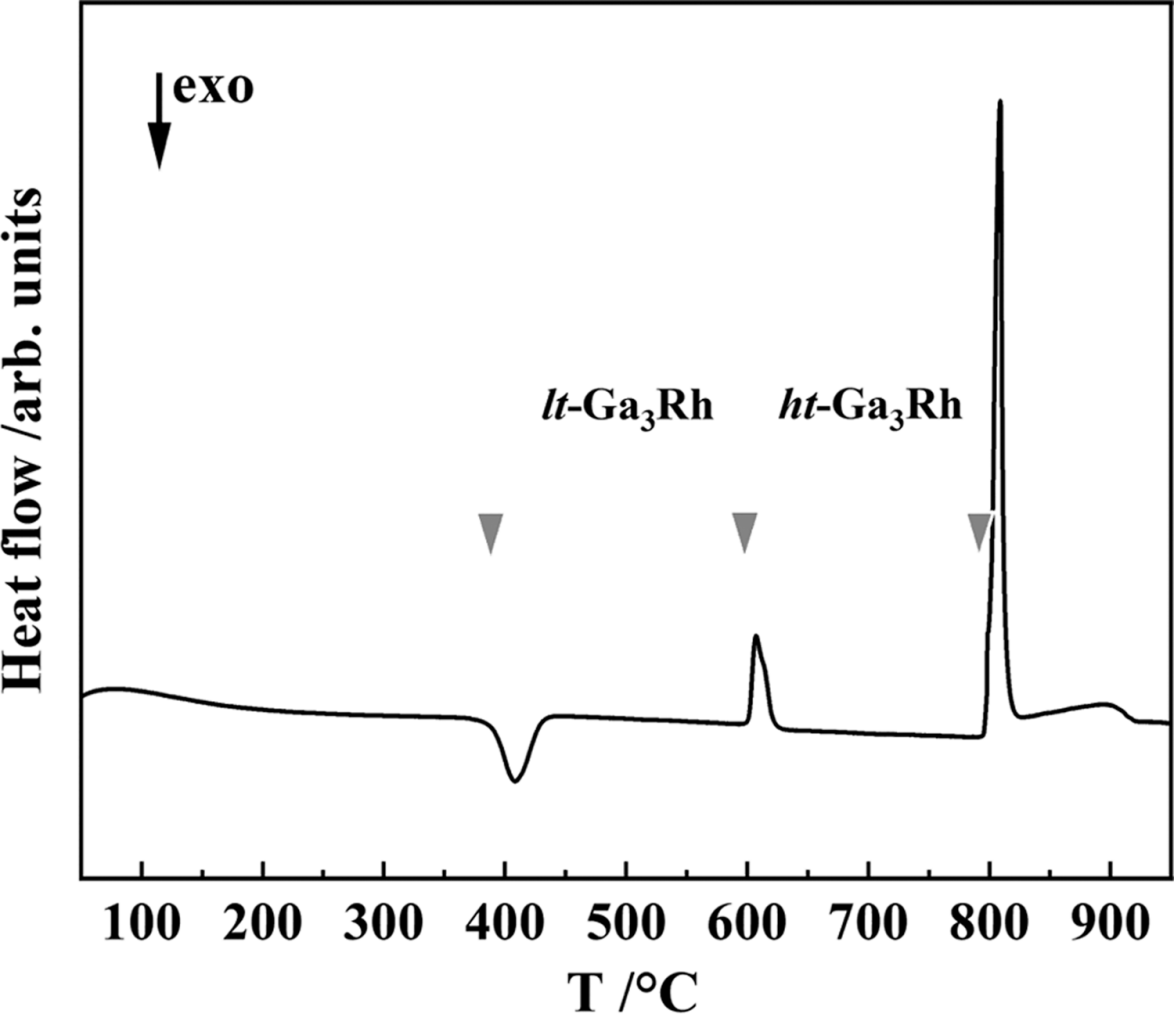
Thermal behavior of the *ht*-Ga_3_Rh sample (obtained by quenching after annealing at 700 °C) upon heating, showing the *ht* → *lt* and *lt* → *ht* transformations at 389(2) and 594(2) °C, respectively, and peritectic decomposition at 792(2) °C of the *ht*-Ga_3_Rh phase.

The sample obtained by quenching after annealing at 700 °C contains virtually single-phase *ht*-Ga_3_Rh and was used to determine the temperature of the *lt* ↔ *ht* phase transition and the stability range of the high-temperature modification. The exothermic effect at 389 °C indicates the *ht*-to-*lt* transformation due to the metastability of the *ht* modification at lower temperatures. The endothermal DSC peaks at 594 and 792 °C observed by heating of *lt*-Ga_3_Rh (Figure 2) correspond to the transformation of the low- into the high-temperature modification and the peritectic decomposition of *ht*-Ga_3_Rh, respectively. The PXRD pattern of the decomposition products shows the presence of Ga_5_Rh_3_.^34^

### Crystal Structure

**Figure 3:**
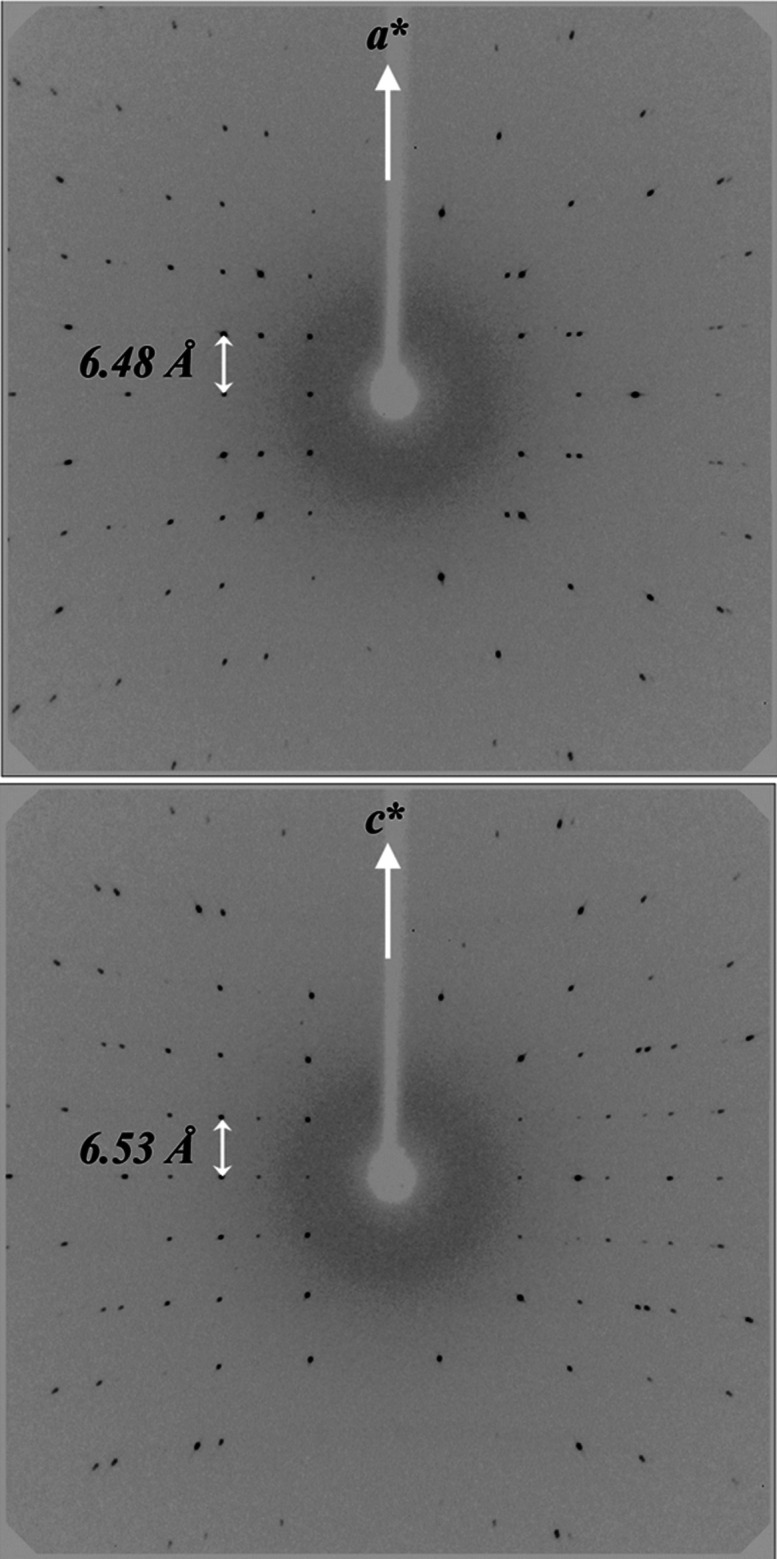
Axial diffraction patterns (Mo Kα radiation) around a* (top) and c* (bottom) for *ht*-Ga_3_Rh.

Appropriate nonregularly shaped crystal of *ht*-Ga_3_Rh fixed with glue at the top of a glass capillary was mounted on a Rigaku AFC7 automatic diffractometer for intensity data collection. Axial diffraction patterns (partial ω-rotation) confirmed tetragonal symmetry with the lattice parameters *a* = *b* ≈ 6.48 and *c* ≈ 6.53 Å. No additional reflections indicating the formation of a superstructure were observed (Figure 3).

Fulfilling the extinction conditions (observed reflections 0*kl* with *k* + *l* = 2*n*, 0*k*0 with *k* = 2*n*, 00*l* with *l* = 2*n*) and considering possible isotypism of *ht*-Ga_3_Rh with Ga_3_Fe,^16^ the centrosymmetric space group *P*4_2_/*mnm* was selected for the crystal structure refinement. Crystallographic information is given in Table 1. Despite the respectable residual value of *R*_F_ = 0.045, the so-obtained model showed two essential drawbacks: (i) unusually large atomic displacement parameters (ADPs) for both gallium positions: *B*_eq_(Ga1) ≈ 3 × *B*_eq_(Rh) and *B*_eq_(Ga2) ≈ 5 × *B*_eq_(Rh) (Table 2) and (ii) relatively large residual densities. Such features were recently observed for the *ht*-Ga_3_Ir phase.^14^ Attempts of refinement in the space groups *P*4_2_*nm* and *P*4̅*n*2 with the same extinction symbol *P-n*- did not improve the model (Tables S2 and S3).

**Table 1:** Crystallographic Information for *ht*-Ga_3_Rh

composition		
from structure refinement	Ga_3_Rh_1_	
from WDXS data	Ga3.000(2)Rh0.990(2)	
Pearson symbol	*tP*16	
molar mass	312.07	
crystal color, shape	gray, prismatic	
crystal dimensions (mm^3^)	0.035 × 0.050 × 0.057	
space group, *Z*	*P*4_2_/*mnm* (no. 136), 4	
lattice parameters (Å)^a^		
*a*	6.4808(2)	
*c*	6.5267(2)	
*V* (10^6^ pm^3^)	274.13(3)	
calculated density (g cm^–3^)	7.56	
diffractometer, detector	Rigaku AFC7. CCD, Saturn724+	
radiation	Mo Kα (λ = 0.71073 Å)	
exposures, steps	900, φ = 0.8°	
absorption correction	multiscan (μ = 36.091 mm^–1^)	
*T*_min_/*T*_max_	0.128/0.283	
2θ_max_; sin θ/λ	86.1°; 0.96	
*hkl* range	–10 < *h* < 12	
	–12 < *k* < 11	
	–12 < *l* < 4	
reflections		
measured	5258	
used in refinement	558	
*R*_(eq)_; *R*_(σ)_	0.039; 0.016	
observation criteria	*F*(*hkl*) > 4σ *F*(*hkl*)	
refinement		
	Ideal IrIn_3_-type Structure	Split Model
parameters	16	27
*R*(*F*), *R*_w_	0.045, 0.050	0.029, 0.033
goodness-of-fit	1.03	1.02
Δρ_min_, Δρ_max_ (e Å^–3^)	–1.3, 2.1	–0.53, 0.91

a Lattice parameters obtained from powder X-ray diffraction data.

**Table 2:** Atomic Positions and Displacement Parameters *B_ij_* [Å^2^] for *ht*-RhGa_3_ (Structure Type In_3_Ir, Space Group *P*4_2_/*mnm*)

atom	Rh	Ga1	Ga2
site	4*f*	4*c*	8*j*
*x*/*a*	0.15736(6)	1/2	0.3525(2)
*y*/*b*	*x*	0	*x*
*z*/*c*	0	0	0.2519(3)
*B*_iso_^a^	0.60(1)	2.28(3)	3.12(3)
*B*_11_	0.56(2)	1.10(4)	2.55(3)
*B*_22_	*B*_11_	2.91(6)	*B*_11_
*B*_33_	0.67(2)	2.82(6)	4.26(6)
*B*_12_	–0.01(1)	1.11(4)	1.38(3)
*B*_13_	0	0	–2.51(4)
*B*_23_	0	0	*B*_13_

a *B*_iso_ = 1/3[*B*_11_a*^2^*a*^2^ + ··· 2*B*_23_b*c*bc cos(α)].

**Figure 4:**
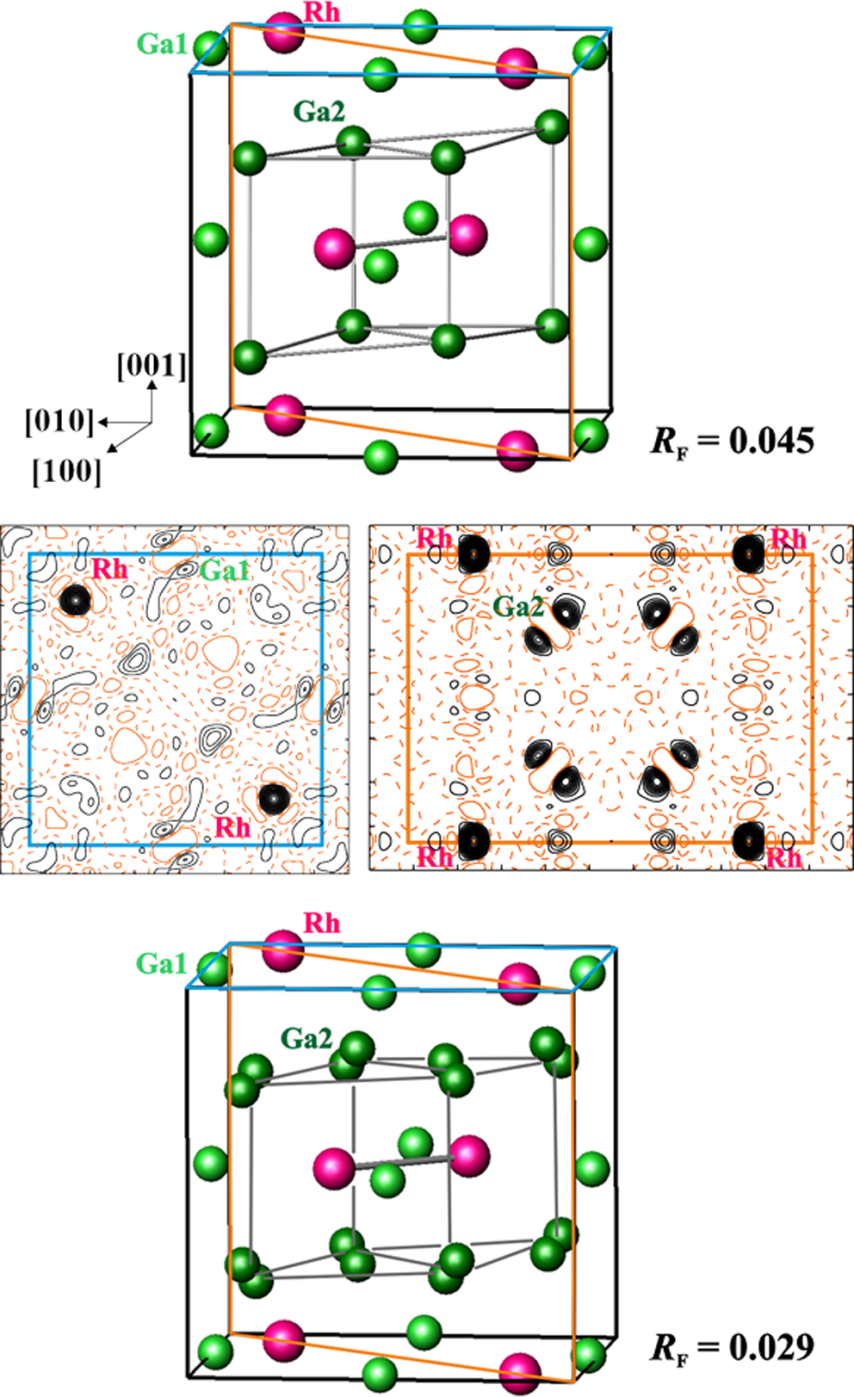
Crystal structure of *ht-*Ga_3_Rh. (Top) IrIn_3_-type model according to Table 2 with (001) and (110) planes (blue and orange, respectively) for calculation of residual density. (Middle) Distribution of the residual density in the (001) and (110) planes (left and right, respectively), revealing disorder of the Ga1 and Ga2 positions. (Bottom) Split model of the crystal structure in the space group *P*4_2_/*mnm* according to Table 3. The [Ga_8_Rh_2_] rhombic prisms in both models are outlined for a better comparison.

Calculation of the residual electron density with isotropic displacement parameters for both gallium species fixed to physically reasonable values revealed undescribed residual density around these positions (Figure 4), which agrees with the form and orientation of the displacement ellipsoids (Figure S4, top). In order to describe the density more completely, split positions for Ga1 and Ga2 were applied (Table 3). This allowed the reduction of the ADPs for these sites and markedly reduced the residual value to *R*_F_ = 0.029 (Table 1). The final values of atomic coordinates are presented in Table 3 (the interatomic distances are given in Table S4).

**Table 3:** Atomic Positions and Displacement Parameters [Å^2^] for *ht*-Ga_3_Rh Crystal Structure (Split Model, Space Group *P*4_2_/*mnm*)

atom	Rh	Ga1	Ga21	Ga22
site	4*f*	16*k*	8*j*	8*j*
*x*/*a*	0.15730(5)	0.5077(7)	0.3409(2)	0.3718(2)
*y*/*b*	*x*	0.0155(4)	*x*	*x*
*z*/*c*	0	0.0157(3)	0.2684(2)	0.2240(3)
Occ.	1.000	0.250	0.568(6)	0.432
*B*_iso_^a^	0.592(7)	1.65(3)	1.72(2)	2.23(3)
*B*_11_	0.567(8)	0.90(3)	1.54(2)	1.91(4)
*B*_22_	*B*_11_	2.09(5)	*B*_11_	*B*_11_
*B*_33_	0.68(1)	1.97(5)	2.08(4)	2.86(7)
*B*_12_	0.000(8)	0.69(4)	0.37(3)	0.66(4)
*B*_13_	0	0.07(11)	–1.00(3)	–1.47(4)
*B*_23_	0	0.42(10)	*B*_13_	*B*_13_

a *B*_iso_ = 1/3[*B*_11_a*^2^*a*^2^ + ··· 2*B*_23_b*c*bc cos(α)].

The presence of two modifications for Ga_3_Rh makes this system comparable to the Ga–Ir one. No dynamic character of the disorder has been found in *ht*-Ga_3_Ir by low-temperature single-crystal diffraction experiments.^14^ This analogy supports the static disorder in *ht*-Ga_3_Rh. An additional hint for this is the relatively large distance of 0.41 Å between the positions Ga21 and Ga22, describing the electron density in the vicinity of Ga2 in the split model.

For further analysis and quantum mechanical calculations, ordered variants of the split model were constructed in the space groups *P*4_2_*nm, P*1̅, and *P*4_2_2_1_2 by reducing the symmetry and using the experimental atomic coordinates from Table 3 in different combinations (Figure 4, cf. also the Experimental Section). Averaging the atomic coordinates of all models yields the ideal structure of the In_3_Ir type (Table 2).

**Figure 5:**
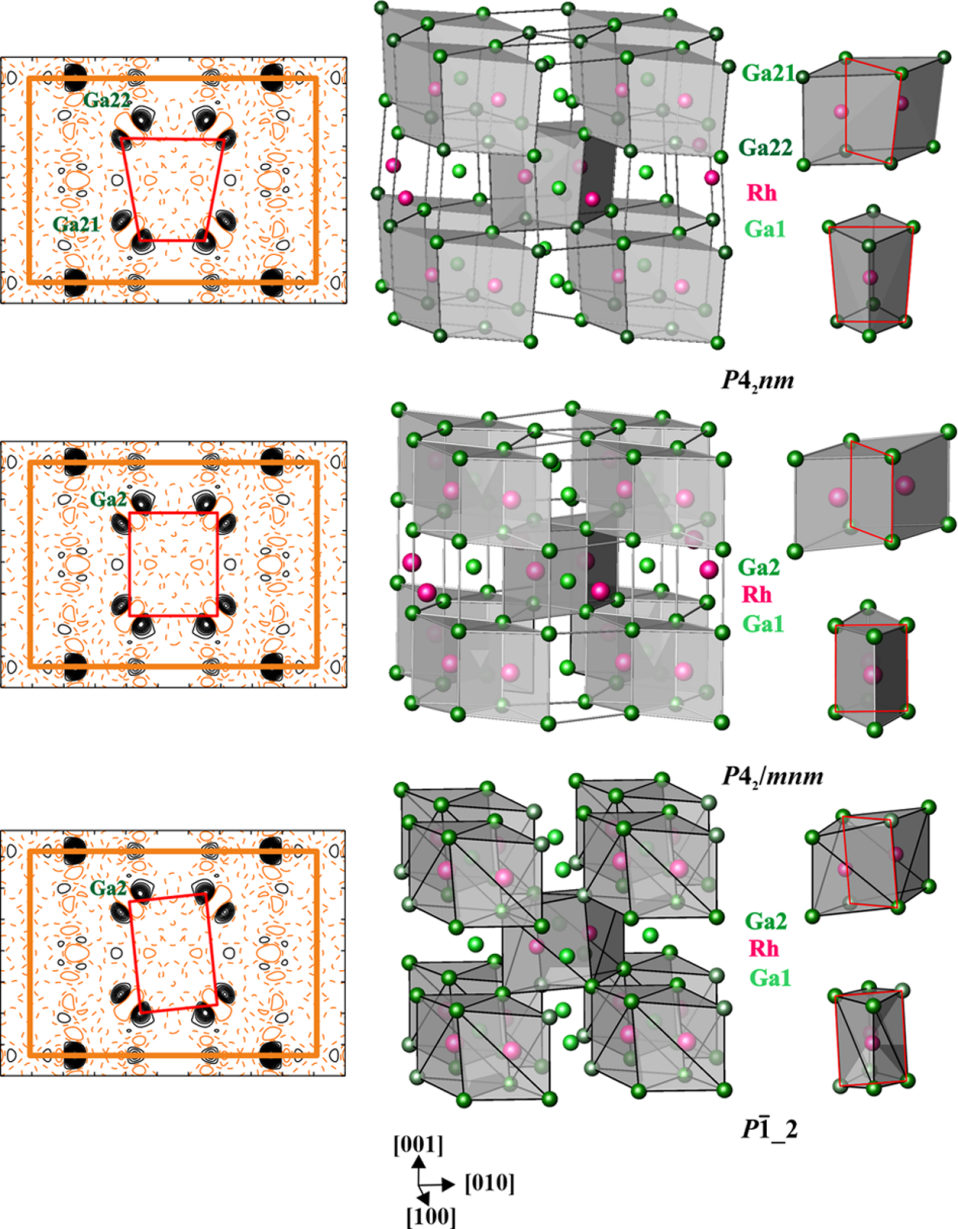
Models of the crystal structure of *ht*-Ga_3_Rh: assignment of the residual density peaks to the atomic sites in different models (left, the atoms of the common quadrilateral face of the trigonal prisms are connected by red lines); [Ga_8_Rh_2_] as the main building block (right) and framework formed by rhombic prisms (middle) in the IrIn_3_-type model (*P*4_2_/*mnm* model, middle), and in the ordered split models (*P*4_2_*nm* model, top, *P*1̅_2 model, bottom).

The crystal structures of the IrIn_3_ type are typically described as the framework built of TM-centered vertex-condensed rhombic prisms (RPs) [Ga_8_TM_2_] or tetra-capped rhombic prisms (TCRPs).^16^ In this representation, the ordered models differ in the kind of distortion of the RPs with respect to the “average” *P*4_2_/*mnm* model (Figure 5). While in the latter, the RPs have all edges either parallel or perpendicular to [001] and the middle quadrangle defined by four Ga2 atoms is a rectangle parallel to [001], the rectangular quadrilaterals of the RPs in the *P*1̅ model 1 (*P*1̅_1) are rotated clockwise or anticlockwise by a small angle around [110] and [11̅0], in the *P*4_2_*nm* model, the middle quadrangles of the RPs have the shape of a trapezium, and in the *P*1̅ model 2 (*P*1̅_2), the middle quadrangles of the RPs have the shape of a parallelogram (cf. split of the Ga2 position in Table 2 to Ga21 and Ga22 in Table 3 and Figure 5, left). In the *P*1̅_1 and *P*4_2_2_1_2 models, the quadrangles are flat or twisted rectangles (Figure S6). Within the RP, most of the shortest heteroatomic distances do not differ essentially between the models (cf. Figure S1, Tables S4 and S5). As an exception, the shortest distance *d*(Ga2–Ga2) changes from 2.65 Å in the *P*4_2_2_1_2 model via 2.64 Å in the *P*1̅_1 model to 2.53 Å in the *P*1̅_2 model and to 2.35 Å in the *P*4_2_*nm* model. The last one is unusually short and represents the test case, whether the “disproportionation” of the Ga2–Ga2 contacts (bridging the Rh–Rh ones) in one short and one long in this model, in comparison with the *P*4_2_/*mnm* one, influences the bonding picture in *ht*-Ga_3_Rh.

### Chemical Bonding

The RPs, completed by four additional Ga1 atoms capping the side faces, form tetra-capped rhombic prisms (TCRPs) filled with two TM atoms (Figure S4, bottom) and are considered as the main building block of the Ga_3_TM compounds. Here, each Ga atom is shared between two TCRPs within the framework.^16^

With the composition  = Ga1_2_Ga2_4_TM_2_, such a block represents two formula units Ga_3_TM. Inside the block, each TM atom is coordinated by two terminal Ga1, four bridging Ga2, two terminal Ga2 ligands, and one further TM atom. In sum, 17 short interatomic distances are counted within the TCRP block: 12 TM–Ga2 (<2.60 Å), 4 TM–Ga1 (<2.46 Å), and 1 TM–TM (<2.89 Å). The next essential distance Ga2–Ga2 perpendicular to [001] (two per TCRP) varies strongly between 2.35 and 3.70 Å, depending on the model (Table S5). In the case of TM = Fe, the total number of 34 valence electrons per TCRP block is just sufficient to realize this model construction by allocation of two electrons to each of the 17 contacts. In this way, the 18-electron rule for Fe atoms is formally satisfied assuming the presence of the Fe**–**Fe bond.^16^ The additional electron contributed by each Rh atom makes the formation of the Rh–Rh bond unnecessary to fulfill the 18-electron rule in *ht*-Ga_3_Rh. Even more, these 2 additional electrons per rhombic prism are expected to fill a Rh–Rh antibonding state, which would be expected to completely cancel out the bonding contributions, if no further mixing with formally unoccupied states or other processes for their dissipation occurred. The complete cancellation of TM–TM (Co–Co) bonding does not take place, as shown from predictive delocalization index calculations for Ga_3_Co, where a combination of mixing with Co(4s,4p) states and certain amounts of Ga2–Ga2 bonding yields a DI decrease of about 30% from 0.42 to 0.30.^11,16^ Proceeding from Ga_3_Fe to Ga_3_Co, the decreased Co–Co bonding, indicated by the DI, is large enough to cause the disappearance of the Co–Co ELI-D basin in Ga_3_Co. Therefore, to study the reasons for the distortion of the rhombic prisms in the crystal structure of *ht*-Ga_3_Rh and the role of the TM–TM bonds, chemical bonding was studied within the position-space electron-localizability approach.^28^

**Figure 6:**
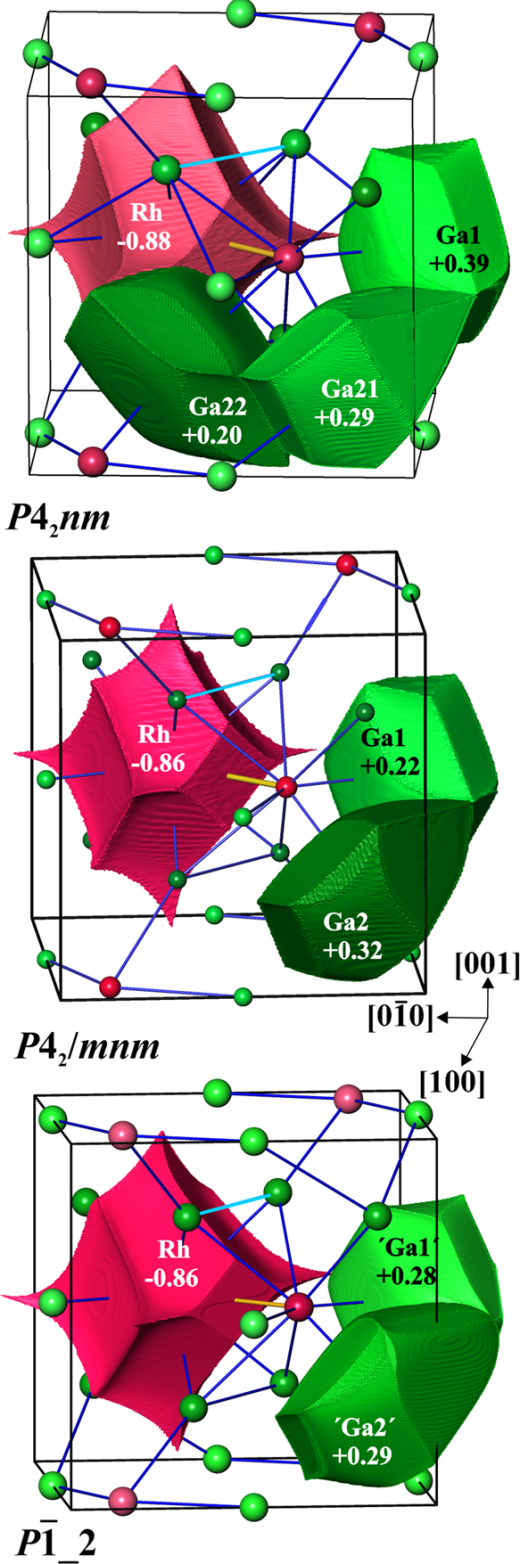
QTAIM atomic shapes and effective charges in three models of the crystal structure of *ht*-Ga_3_Rh.

In all models of the crystal structure of *ht*-Ga_3_Rh, the shapes of the QTAIM atomic basins are very similar (Figures 6 and S8). The atomic shapes of Rh and both gallium atoms are plane between atoms with the same or similar charge and concave from the negatively toward positively charged species. The only essential difference is the size of the flat surfaces of the Rh atom toward the neighboring Rh ligand. In the low-symmetry models, the size of this face is smaller (Figures 6 and S8), which may be considered as a hint of a weakening Rh–Rh interaction. This is confirmed by quantitative analysis of the surface for the QTAIM Rh atoms (Figure S8). Furthermore, the electron density value at the midpoint of the Rh–Rh contact in the *P*4_2_/*mnm* model with 0.39 e Å^–3^ is essentially larger than 0.29 e Å^–3^ in the *P*1̅_2 model or 0.28 e Å^–3^ in the *P*4_2_*nm* model. These differences are observed at the same distance of 2.88 Å in all models (Table S7). The charge transfer indicated by the effective QTAIM atomic charges is very similar in all models (Figure 6). Rhodium plays the role of the anionic component, justifying the way in which the chemical formula of the Ga_3_TM compounds is written, as suggested in the section Introduction. This direction of charge transfer is consistent with electronegativity difference according to the Pauling scale (EN(Rh) = 2.28, EN(Ga) = 1.81^35^) but not according to the Allen one (EN(Rh) = 1.56, EN(Ga) = 1.76^36−38^). The same charge transfer direction is also found for Ga_3_Fe^16^ and all of the other Ga_3_TM compounds with this structure type.^11^ Neither Allen’s nor Pauling’s or Allred-Rochow’s EN is completely consistent with the clear trend found.^11^ It is supposed to be the result of connectivity-dominated topological charge control and not by free atoms’ property differences. Such behavior is opposite to the synergetic topological charge stabilization concept, where the buildup of negative atomic charge is supposed to be simultaneously supported by the higher electronegativity of the atom at this site.^39,40^ Similar behavior for TMB_2_ and TMB_4_ compounds has been reported in the framework of Mulliken population analysis as well.^41^ It has been argued that it is more realistic to consider the charge transfer between the two homoatomic sublattices, TM and B, respectively, instead of between the free separate atoms characterized by their atomic electronegativities.^39,40^

Next, the difference between the IrIn_3_-type *P*4_2_/*mnm* model and its distorted variants is found in the QTAIM charge size at the gallium positions Ga1 and Ga2 (or its equivalents in low-symmetry models, Figures 6 and S8). Considering the tendency of Rh to accumulate electrons, from the two-coordination of Ga1 by Rh and the three-coordination of Ga2 by rhodium, one expects a larger charge for the Ga2. This is found for the *P*4_2_/*mnm* model but not for the low-symmetry models. Despite all of the differences described above, in sum, the consideration of only the charge transfer does not offer arguments to explain the unique distortion of the RP framework for *ht*-Ga_3_Rh.

Further information about the chemical bonding is obtained from the combined analysis of electron density and the electron-localizability indicator. In all models of *ht*-Ga_3_Rh, the penultimate shells of Ga atoms are spherical (nonstructured), while the penultimate shell (4th) of the rhodium atoms is clearly structured, indicating its contribution (predominantly 4d orbitals) to the bonding situation in the valence region (cf. Figure S4 for *P*4_2_/*mnm*, *P*4_2_*nm*, and *P*1̅_2 models).^28,42,43^

**Figure 7:**
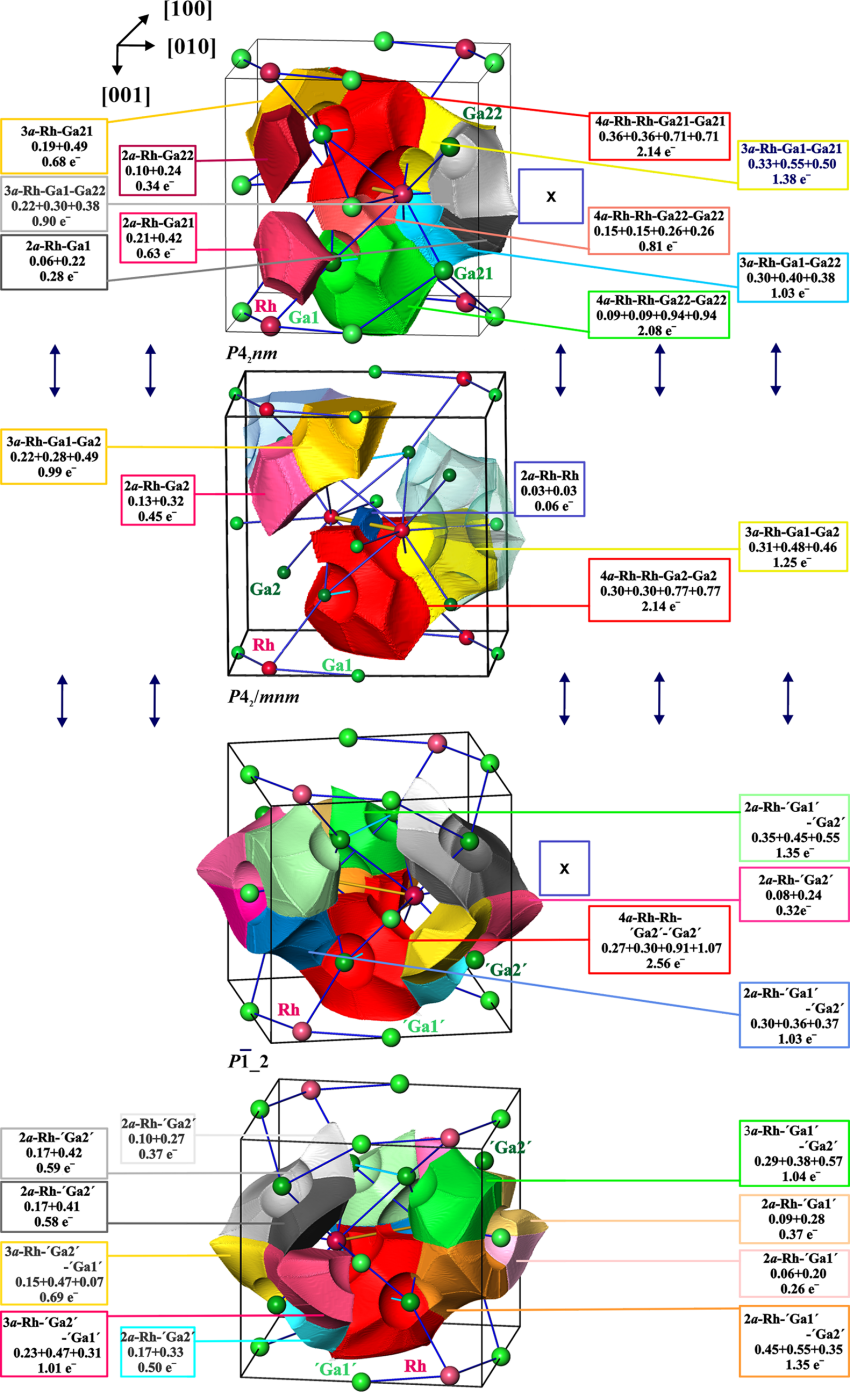
Bond basins, their atomicity, and electron populations in *P*4_2_*nm* (top), *P*4_2_/*mnm* (middle), and *P*1̅_2 (bottom) models of the crystal structure of *ht*-Ga_3_Rh. For other ordered models, see Figure S5. The labels Ga21, Ga22 (top panel) and “Ga1”, “Ga2” (bottom panel) denote the positions derived from initial Ga1, Ga2 (middle panel) due to the symmetry reduction. The frame’s color code is the same as that for the bond basins in the middle panel. The bond basin populations derived from that in the pristine *P*4_2_/*mnm* model are located in the same columns right and left.

The local maxima of ELI-D in the valence region visualize different types of bonds. Integration of the electron density within the bond basins yields their electronic populations (Figures 7 and S10). Analysis of the ELI-D/QTAIM basin intersections results in the total and the effective bond basin atomicity,^28^ i.e., the number of atoms that significantly contribute to the bond basin population. In the case of *ht*-Ga_3_Rh, this information shows that the bonding does not involve only two atoms (like in the classical 2-center bonding scenario) for virtually all kinds of short interatomic contacts.

Five different types of bonds are found by analysis of the corresponding ELI-D valence basins in the *P*4_2_/*mnm* model (Figure 7, middle). With 13 bond basins per TCRP, the total number is less than 17 obtained from counting the nearest-neighbor contacts because several ELI-D bond basins represent 3- and 4-atomic bonding situations.

ELI-D/ED basin intersection analysis shows that rhodium participates in all of the bond basins. Despite negative QTAIM charge, the latter makes minor contributions to all four types of heteroatomic bond basins, and the major contributions there are coming from gallium atoms. The negative QTAIM charge of rhodium originates from the large number of bonds where rhodium is participating. These contributions sum to 3.01 electrons per rhodium atom being more than formally one electron in the last shell in the [Kr] 4d^8^5s^1^ configuration. The free Rh atom was found to display no real-space boundary between the fourth and fifth shells (in the scalar relativistic ELI-D framework^44^). Thus, the 3.01 electrons in the valence (5th) shell of Rh in *ht*-Ga_3_Rh is a chemical bonding effect related also to the bonding participation of the 4d electrons as revealed by the structuring of the penultimate shell of Rh in the ELI-D distribution (Figure S9) and spreading their orbital densities into the valence region.^35^ The sum of the Rh valence shell and inner shell electrons yields 45.85 electrons, which is in full agreement with rhodium’s QTAIM charge of −0.86 (Figure 6). For gallium atoms, 3.04 (Ga1) and 2.89 (Ga2) electrons were found in the bond basins (in the *P*4_2_/*mnm* model). Additional inclusion of the exact Ga core–shell underpopulations displayed by the actual ELI-D distribution for Ga species in each model, which vary between 0.2 and 0.3 electrons (consistent with the free-atom value of 0.368 electrons),^44^ yields the finally obtained small positive QTAIM charges of gallium species in all models (Figure 6).

Homoatomic bonding between rhodium atoms is topologically indicated in the *P*4_2_/*mnm* model by the presence of an ELI-D basin centered at the midpoint of the Rh–Rh internuclear line, albeit at a very low bond basin population of 0.06 e^–^.

Due to the lower symmetry, the number of different bond types in the low-symmetry ordered models is larger than in the *P*4_2_/*mnm* one (Figures 7 and S10). All heteroatomic bonds, known from the *P*4_2_/*mnm* model, are present in several variations also in the low-symmetry models. Here also, rhodium participates in all heteroatomic bonds as a minor partner. The characteristic difference to these models is the absence of the dedicated bond basin between the Rh atoms. It is only included in the 4*a*-Rh–Rh–Ga2–Ga2 bond basin (red basin in the bottom panels of Figures 7 and S10), but here the Ga2 contributions are the majority ones. This means that these two Ga2–Ga2 contacts within the TCRP should be included into the conceptual electron counting (2-electron bonds). That gives 19 bonds, which are not realizable with 36 available valence electrons. However, it can be realized if the one topologically absent Rh–Rh bond would be excluded (18 bonds). This agrees well with the ELI-D picture of the bonding (Figure 7, bottom panels).

In total, the bonding picture in *ht*-Ga_3_Rh can be understood as a superposition of the *P*4_2_*nm* and *P*1̅_2 models (mainly). While in Ga_3_Fe, the Fe–Fe dumbbell bonds were shown to play a key role in the bonding picture and formation of rhombic prisms, the absence of the TM–TM bonds in *ht*-Ga_3_Rh seems to be only one of the essential reasons for the experimentally observed distortion of the RPs. The most important role plays the formation of the short Ga2–Ga2 bonds bridging the Rh–Rh contacts. The decrease of the Rh–Rh bonding coupled with the increase of the Ga2–Ga2 bonding in the sense of a cooperative effect is the clear signature of the experimentally observed disorder. Furthermore, it points to the structure and bonding scenario in the low-temperature modification. The two essential bonding features of *ht*-Ga_3_Rh—the absence of the Rh–Rh contacts and the presence of the Ga–Ga bonds—are pronounced in the low-temperature modification *lt*-Ga_3_Rh.^33^

**Figure 8:**
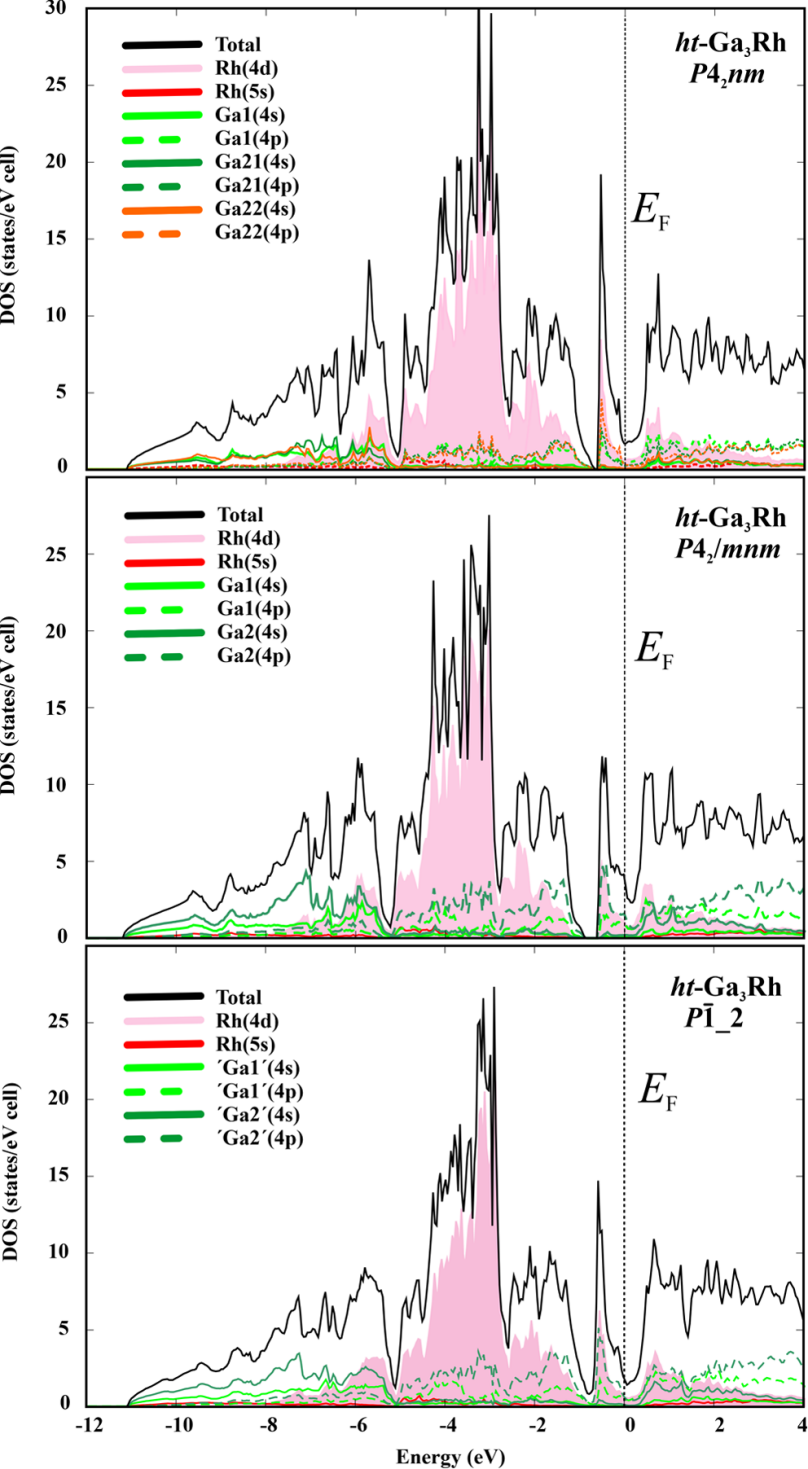
Calculated electronic density of states for *ht*-Ga_3_Rh models with the space groups *P*4_2_*nm* (top), *P*4_2_/*mnm* (middle), and *P*1̅___2 (bottom).

The differences in atomic arrangements and bonding pictures in all models of *ht*-Ga_3_Ir are reflected only by very specific variations in their calculated electronic densities of states (DOS), while the overall pictures are very similar (Figure 8). The DOS for the *P*4_2_/*mnm* model (Figure 8, middle) agrees well with the previous calculations made with other codes and density functional theory (DFT) functionals.^11,13^ The calculated DOS below the Fermi level can be divided into three regions. The low-energy one (*E* < −5.3 eV) is separated from the next one by a deep dip and is composed mainly of the contributions of the s states of Ga with a small participation of Rh(d). The middle energy region (−5.3 < *E* < −0.8 eV) is mainly formed by the Rh(d) states with an admixture of p states of Ga and is separated from the third one by a gap of around 0.2 eV. This gap represents the fundamental band gap in the Ga_3_TM compounds with 17 valence electrons per formula unit. Above this gap (−0.6 eV < *E* < *E*_F_), the third region with an electronic population of 4 valence electrons per unit cell, i.e., one per formula unit is located. This region represents the difference between the Fe and Rh in valence electron number, which was discussed above for the chemical bonding picture. The region shows a sharp peak formed by Rh(d) and Ga2(p) states. At the Fermi level, the DOS shows a pronounced dip and is controlled by Ga(p) and Rh(d) contributions. The essential differences between the models are the smaller pseudogaps and the sharper peaks in the DOS in the region above the fundamental gap −0.8 eV as well as the smaller DOS at the Fermi level in the *P*1̅_2 and *P*4_2_*nm* models, and the DOS curvature at the Fermi level. A striking feature of DOS for *ht*-Ga_3_Rh is—in contrast to Ga_3_Ir^11,13^ but similar to Ga_3_Co^11^—the nonzero density of states at the Fermi level, which is important for the understanding of the thermoelectric properties.

### Thermoelectric Properties

**Figure 9:**
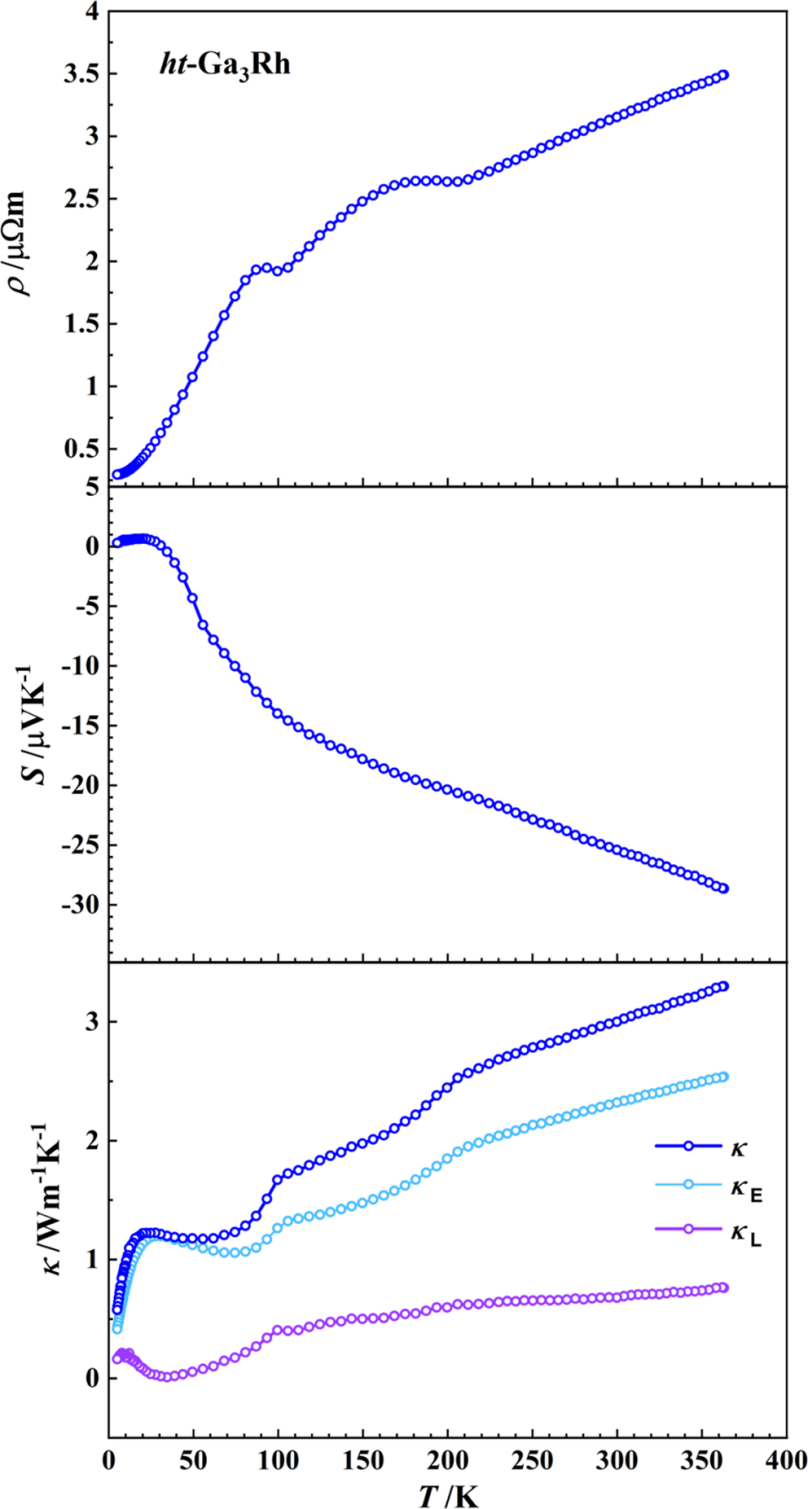
Temperature dependence of thermoelectric properties of *ht*-Ga_3_Rh: (top) electrical resistivity ρ; (middle) Seebeck coefficient *S*; (bottom) thermal conductivity κ with the electronic (κ_E_) and lattice contribution (κ_L_).

Measurements on compacted and sintered samples show the characteristic temperature dependence of the electrical resistivity, indicating a metal-like behavior of *ht*-Ga_3_Rh (Figure 9, top) in agreement with the DOS calculations above and previous results.^11^ The nature of the shoulders at 100 and 200 K is not clear. These effects are only weakly reflected by the temperature behavior of the Seebeck coefficient but are clearly visible in the thermal conductivity. In addition, clear effects are observed in the temperature dependence of the specific heat (Figure S6). This may suggest some structural rearrangements. Their nature, in particular the connection of these features with the fact that the measurement was made on the sample of the high-temperature modification *ht*-Ga_3_Rh, which is thermodynamically metastable under measurement conditions, should be the subject of further studies.

The negative Seebeck coefficient (Figure 9, middle) shows that electrons are the charge carriers, which agrees with the negative value of ∂DOS/∂E at the Fermi level (Figure 8). Around room temperature, a Seebeck voltage of −25 μV K^–1^ is induced, being in the range of typical values for metals, in good agreement with the calculated DOS (Figure 8) as well.

The thermal conductivity κ increases with the temperature, revealing the usual behavior for metallic materials. The lattice (phonon) contribution κ_L_ is obtained by subtracting from the total thermal conductivity its electronic part calculated from the Wiedemann–Franz law as κ_E_ = *L*σ*T*, where *L* = 2.44 × 10^–8^ W Ω K^–2^ is the Lorenz number and σ is the measured electrical conductivity at temperature *T*. The obtained low value of κ_L_ ≈ 0.7 W m^–1^ K^–1^ at 300 K is comparable to that of *ht*-Ga_3_Ir (≈ 0.7 W m^–1^ K^–1^ at 300 K^14^) and is lower than that of other known Ga_3_TM compounds being usually above 3 W m^–1^ K^–1^ at 300 K.^4^ This behavior can be related to the increased bonding inhomogeneity (bonding complexity) reflected by a larger number of different bond kinds in *ht*-Ga_3_Rh and *ht*-Ga_3_Ir compared to other Ga_3_TM, e.g., Ga_3_Fe.^16^ From the structural point of view, the bonding inhomogeneity goes along with the experimentally observed positional disorder (cf. the split model above). Similar observations were made on intermetallic clathrates,^45^ where the appearance of additional Ba−TM bonds leads to essential reduction of the lattice thermal conductivity and the thermoelectric goodness-of-fit value above 1. In the Zintl phases BaCu_2_Te_2_ and BaZn_2_Sb_2_^46^ and other thermoelectric materials,^47^ the increase of bonding complexity is observed solely along one of the crystallographic directions, which results in the marked anisotropy of the (lattice) thermal conductivity.

## Conclusions

The reinvestigation of the intermetallic compound Ga_3_Rh reveals the existence of two temperature-dependent modifications. The high-temperature modification *ht*-Ga_3_Rh is stable between 594 and 792 °C. Its crystal structure can be understood as a variant of the IrIn_3_ prototype with a framework formed by distorted vertex-connected rhombic prisms  or tetra-capped vertex-connected rhombic prisms  around two Rh atoms. The distortion arises from the disorder of Ga2 species in the middle planes of the rhombic prisms, which is described with ordered low-symmetry models, which, on average, yield the pristine high-symmetry structure of the IrIn_3_ type. Position-space analysis of chemical bonding in the ordered model reveals the absence of the conceptually countable TM–TM bonds and the formation of homoatomic Ga2–Ga2 bonds besides the heteroatomic Ga–Rh ones within the rhombic prisms as essential reasons for the experimentally observed distortion of the rhombic prisms. In agreement with the calculated electronic structure, *ht*-Ga_3_Rh shows a bad-metal-like temperature dependence of electrical conductivity with electrons as charge carriers. The increased bonding complexity (large number of different bond types) results in low lattice thermal conductivity (κ_L_) of *ht*-Ga_3_Rh being similar to that of *ht*-Ga_3_Ir but significantly lower in comparison with other Ga_3_TM compounds.

## Supporting Information Available

Crystallographic description of the ordered models, refinements results in the low-symmetry groups, tables of interatomic distances, and results of bonding analysis in low-symmetrical models (PDF)
