# Supplementary material for: On Chemical Bonding in ht-Ga3Rh and Its Effect on Structural Organization and Thermoelectric Behavior
Source: Inorg Chem. 2024 Jun 14;63(26):12156–66. doi: 10.1021/acs.inorgchem.4c01280 (PMC11220751; doi:10.1021/acs.inorgchem.4c01280)
Supplement: Supplementary file 1 — ic4c01280_si_001.pdf [file ic4c01280_si_001.pdf]

Supporting Information

On chemical bonding in *ht*-Ga_3Rh and its effect on structural organisation and thermoelectric behaviour

Raúl Cardoso-Gil[1], Mitja Krnel[1], Frank R. Wagner[1], Yuri Grin[1*]*

[1] Max-Planck-Institut für Chemische Physik fester Stoffe, Nöthnitzer Str. 40, 01187 Dresden, Germany

*Raul Cardoso-Gil (raul.cardoso@cpfs.mpg.de); Yuri Grin (grin@cpfs.mpg.de)

Table of contents

Table S1. Ordered models of *ht*-Ga_3Rh used for bonding analysis.

Table S2. Crystallographic data for *ht*-Ga_3Rh in the space groups $P4_2nm$ and $P\bar{4}n2$.

Table S3. Atomic coordinates and displacement parameters [Å^2] in *ht*-Ga_3Rh (space groups $P4_2nm$ and $P\bar{4}n2$).

Figure S1. Crystal structure of *ht*-Ga_3Rh (space group $P4_2/mnm$.

Table S4. Interatomic distances in the crystal structure of *ht*-$RhGa_3$ (ideal $InGa_3$-type and split model).

Figure S2. Ordered models of the crystal structure of *ht*-$RhGa_3$.

Table S5. Selected interatomic distances in the models $P4_2nm$, $P\bar{1}$_1, $P\bar{1}$_2 and $P4_22_12$ of the crystal structure of *ht*-Ga_3Rh.

Figure S3. QTAIM atoms' basins and populations in the ordered models of *ht*-$RhGa_3$.

Figure S4. ELI-D distribution in the (001) and (110) planes in three models of the crystal structure of *ht*-Ga_3Rh.

Figure S5. Bond basins, their atomicity and electron populations in low-symmetrical models.

Figure S6. Specific heat C_P *versus* temperature for *ht*-Ga_3Rh.

Table S1. Ordered models used for bonding analysis in of *ht*-Ga_3Rh

Model $P4_2nm$: space group $P4_2nm$, *a* = 6.4808 Å, *c* = 6.5297 Å; Rh in 4(*c*) *xxz*, *x* = 0.15729, *z* = 0.0; Ga1 in 4(*b*) ½0*z*, *z* = 0.0; Ga21 in 4(*c*) *xxz*, *x* = 0.34085, *z* = 0.26842; Ga22 in 4(*c*) *xxz*, *x* = 0.37179, *z* = 0.77603.

Model P$\bar{1}$_1: space group $P\bar{1}$, *a* = 6.4808 Å, *c* = 6.5297 Å; Rh1 in 2(*i*) *xyz*, *x* = 0.1573, *y* = 0.1573, *z* = 0.0; Rh2 in 2(*i*) *xyz*, *x* = 0.6573, *y* = 0.3427 *z* = 0.5; Ga11 in 1(*d*) ½00; Ga12 in 1(*c*) 0½0; Ga13 in 1(*f*) ½0½; Ga14 in 1(*g*) 0½½; Ga21 in 4(*i*) *xyz*, *x* = 0.3409, *y* = 0.3409, *z* = 0.2684; Ga22 in 4(*i*) *xyz*, *x* = 0.8409, *y* = 0.1591, *z* = 0.7684; Ga23 in 4(*i*) *xyz*, *x* = 0.6282, *y* = 0.6282, *z* = 0.2240; Ga24 in 4(*i*) *xyz*, *x* = 0.1282, *y* = -0.1282, *z* = 0.7240.

Model P$\bar{1}$_2: space group $P\bar{1}$, *a* = 6.4808 Å, *c* = 6.5297 Å, Rh1 in 2(*i*) *xyz*, *x* = 0.1573, *y* = 0.1573, *z* = 0.0; Rh2 in 2(*i*) *xyz*, *x* = 0.6573, *y* = 0.3427 *z* = 0.5; Ga11 in 1(*d*) ½00; Ga12 in 1(*c*) 0½0; Ga13 in 1(*f*) ½0½; Ga14 in 1(*g*) 0½½; Ga21 in 4(*i*) *xyz*, *x* = 0.3525, *y* = 0.3525, *z* = 0.2519; Ga22 in 4(*i*) *xyz*, *x* = 0.8525, *y* = 0.1475, *z* = 0.7719; Ga23 in 4(*i*) *xyz*, *x* = 0.6282, *y* = 0.6282, *z* = 0.2240; Ga24 in 4(*i*) *xyz*, *x* = 0.1282, *y* = -0.1282, *z* = 0.7240;

Model $P4_22_12$: space group $P4_22_12$, *a* = 6.4808 Å, *c* = 6.5297 Å; Rh in 4(*c*) *xx*0; *x* = 0.15729; Ga1 in 4(*f*) ½0z, z = 0.0; Ga2 in 8(*g*) *xyz*, *x* = 0.34085, *y* = 0.37179, *z* = 0.26842.

Table S2. Crystallographic data for *ht*-Ga_3Rh in the space groups $P4_2nm$ and $P\bar{4}n2$. Lattice parameter are obtained from powder x-ray diffraction data, chemical composition from WDXS analysis.

Composition	$Ga_{3.000(2)}Rh_{0.990(2)}$	
Molar mass	312.07	
Crystal color, shape	Gray, prismatic	
Crystal dimensions (mm^3)	0.035 × 0.050 × 0.057	
Lattice parameters (Å); Z	a = 6.4808(2) c = 6.5267(2); 4	
V (10^6 pm^3), ρ (g cm^{-3})	274.13(3), 7.561(1)	
Diffractometer, detector	Rigaku AFC7. CCD, Saturn724+	
Radiation	Mo $K\alpha$ (λ = 0.71073 Å)	
Exposures, steps	900, φ = 0.8°	
Absorption correction	Multi-scan (μ = 36.091 mm^{-1})	
T_{min}/T_{max}	0.128 / 0.283	
$2\theta_{max}$; $\sin\theta/\lambda$	86.1°; 0.96	
hkl range	$–10 < h < 12$<br>$–12 < k < 11$<br>$–12 < l < 4$	
Measured reflections	5601	
Refinement	Full-matrix least-squares on F^2	
Observation criteria	$F(hkl) > 4\sigma F(hkl)$	
Space group	$\boldsymbol{P4_2nm}$ (No. 102)	$\boldsymbol{P\bar{4}n2}$ (No. 118)
Reflections used	722	908
$R_{(eq)}$; $R_{(sigma)}$	0.035; 0.019	0.038, 0.022
Parameters	21	19
$R(F)$, Rw,	0.039, 039	0.043, 0.046
Goodness of fit	1.62	1.02
$\Delta\rho_{min}$, $\Delta\rho_{max}$ (e $Å^{-3}$)	-0.53, 1.05	-1.49, 1.85

Table S3. Atomic coordinates and displacement parameters [Å^2] for crystal structure of *ht*-$RhGa_3$ in the space groups $P4_2nm$ and $P\bar{4}n2$. $B_{iso/eq}$ = 1/3[B_{11} a^{*2} a^2 + ... 2B_{23} b^* c^* b c cos(α)].

	Space group $P4_2nm$				Space group $P\bar{4}n2$		
Atom	Rh	Ga1	Ga2	Ga3	Rh	Ga1	Ga2
Site	4*c*	4*b*	4*c*	4*c*	4*g*	4*e*	8*i*
x/a	0.15731(5)	1/2	0.8572(5)	0.8489(4)	0.34263(5)	0	0.6487(3)
y/b	*x*	0	–*x* + 1	–*x* + 1	–*x* + 1/2	0	0.1463(4)
z/c	–0.02(3)	0.48(3)	0.24(3)	0.74(3)	1/4	0.252(1)	0.0020(2)
Occ.	1.0	1.0	1.0	1.0	1.0	1.0	1.0
$B_{iso/eq}$	0.594(7)	2.25(3)	3.96(10)	2.36(6)	0.600(7)	2.28(4)	3.14(4)
B_{11}	0.561(9)	2.86(5)	3.08(10)	2.02(6)	0.57(1)	1.12(3)	1.96(7)
B_{22}	B_{11}	1.10(3)	B_{11}	B_{11}	B_{11}	2.88(5)	3.22(9)
B_{33}	0.66(1)	2.79(5)	5.7(2)	3.05(14)	0.67(2)	2.82(4)	4.24(4)
B_{12}	–0.018(9)	–1.10(3)	–1.71(10)	–1.06(6)	0.008(8)	–1.11(3)	1.40(2)
B_{13}	–0.14(2)	0	3.42(13)	–1.71(7)	0.08(3)	0	2.30(6)
B_{23}	B_{13}	0	$–B_{13}$	$-B_{13}$	B_{13}	0	2.73(7)

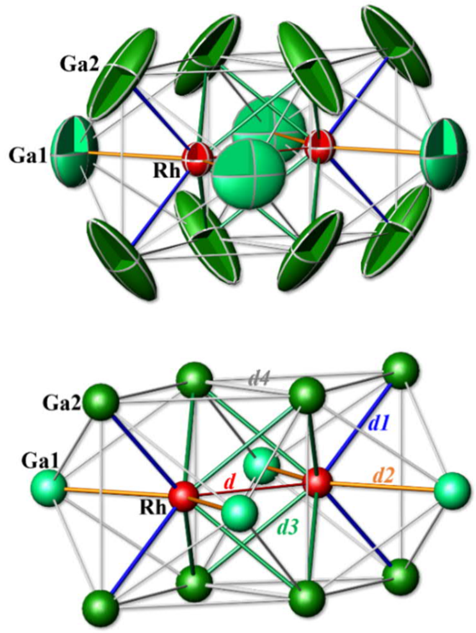

Figure S1. Crystal structure of *ht*-Ga_3Rh (space group $P4_2/mnm$): (top) Atomic displacement ellipsoids (drawn with 99 % probability). (bottom) Notation of interatomic distances in the main building block - TCRP [$Ga2_8Ga1_4Rh_2$], cf. Tables S5, S7.

Table S4. Interatomic distances in the crystal structure of *ht*-$RhGa_3$ in the space group $P4_2/mnm$ ideal $IrIn_3$-type model and split model. Lattice parameters a = 6.4808(2) Å, c = 6.5267(2) Å obtained from powder x-ray diffraction data were used in all models.

$P4_2/mnm$ model ($IrIn_3$-type)			$P4_2/mnm$ split model			
Atoms		d /Å	Atoms			d /Å
Rh -	2×Ga2	2.429(1)	Rh -	2× {	Ga21	2.429(1)
	2×Ga1	2.4436(4)			Ga22	2.450(2)
	4×Ga2	2.555(1)		2× {	Ga1	2.446(3)
	1×Rh	2.884(1)			Ga1	2.451(3)
				4× {	Ga21	2.547(1)
					Ga22	2.589(2)
				1×	Rh1	2.8832(5)
Ga1 -	2×Rh	2.4436(4)	Ga1 -	2× {	Rh	2.446(2)
	4×Ga2	2.959(1)			Rh	2.451(3)
	4×Ga2	2.972(1)		1× {	Ga21	2.742(3)
					Ga22	2.998(3)
				1× {	Ga22	2.820(3)
					Ga21	2.887(3)
				1× {	Ga21	2.853(3)
					Ga22	3.119(3)
				1× {	Ga21	2.893(3)
					Ga22	3.130(3)
				1× {	Ga22	2.925(3)
					Ga21	2.998(3)
				1× {	Ga22	2.960(3)
					Ga21	3.003(3)
				1× {	Ga21	3.009(3)
					Ga22	3.246(3)
				1× {	Ga22	3.059(3)
					Ga21	3.120(3)
Ga2 -	1×Rh	2.429(1)	Ga21 -	1×	Rh	2.429(1)
	2×Rh	2.555(1)		2×	Rh	2.547(1)
	1×Ga2	2.704(1)		1× {	Ga22	2.649(2)
	2×Ga1	2.959(1)			Ga21	2.916(1)
	2×Ga1	2.972(1)		2× {	Ga1	2.742(3)
	(1×Ga2)	3.239(2)			Ga1	2.853(3)
					Ga1	2.893(3)
					Ga1	2.998(3)
				2× {	Ga1	2.887(3)
					Ga1	3.003(3)
					Ga1	3.009(3)
					Ga1	3.120(3)
				(1× {	Ga21)	3.023(2)
					Ga22)	3.226(2)

Ga22 -	1×	Rh	2.450(3)
	2×	Rh	2.589(2)
	1× {	Ga22	2.350(2)
		Ga21	2.649(2)
	2× {	Ga1	2.820(3)
		Ga1	2.925(3)
		Ga1	2.960(3)
		Ga1	3.059(3)
	2× {	Ga1	2.998(3)
		Ga1	3.119(3)
		Ga1	3.130(3)
		Ga1	3.246(3)
	(1× {	Ga22)	2.924(3)
		Ga21)	3.226(3)

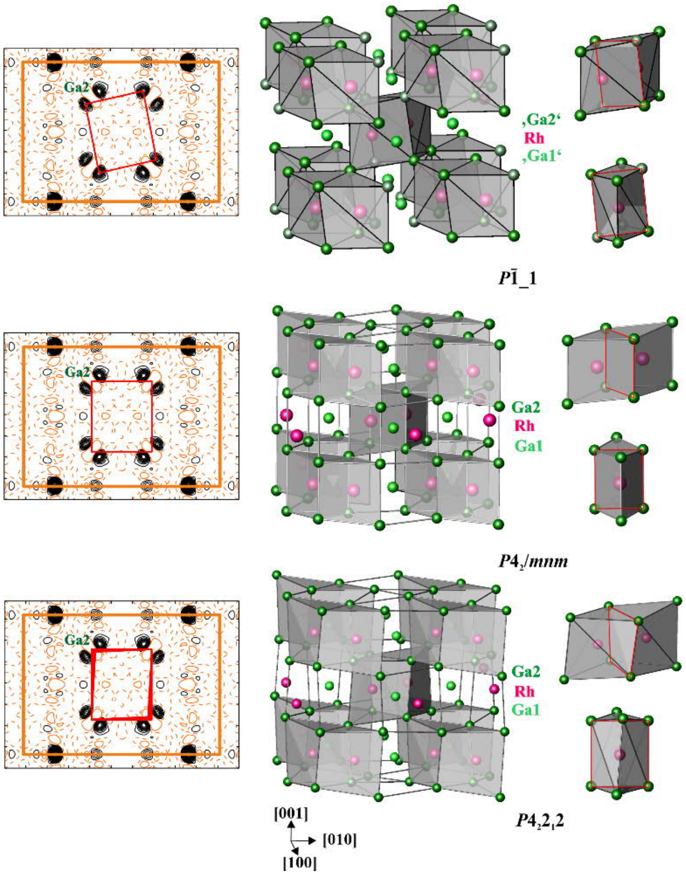

Figure S2. Ordered models of the crystal structure of ht-$RhGa_3$.

Table S5. Selected interatomic distances in the models $P\bar{1}$_1, $P\bar{1}$_2, $P4_2nm$ and $P4_22_12$ of the crystal structure of *ht*-Ga_3Rh. Lattice parameters a = 6.4808(2) Å, c = 6.5267(2) Å obtained from least-squares refinement of powder X-ray diffraction data were used in all models.

$P\bar{1}$_1		
Atoms		*d* /Å
Rh1–	1× Ga3	2.429
	1× Ga1	2.444
	1× Ga2	2.444
	1× Ga5	2.450
	1× Ga4	2.547
	1× Ga6	2.589
	1× Rh1	2.883
Rh2–	1× Ga3	2.429
	1× Ga1	2.444
	1× Ga2	2.444
	1× Ga5	2.450
	1× Ga4	2.547
	1× Ga6	2.589
	1× Rh1	2.883
Ga1–	2× Rh1	2.444
	2× Ga4	2.869
	2× Ga5	2.938
Ga2 –	2× Rh1	2.444
	2× Ga4	2.869
	2× Ga5	2.938
Ga3 –	1× Rh1	2.429
	2× Rh2	2.547
	1× Ga5	2.649
	1× Ga7	2.869
	1× Ga8	2.869
Ga4 –	1× Rh2	2.429
	2× Rh1	2.547
	1× Ga6	2.649
	1× Ga1	2.869
	1× Ga2	2.869
Ga5 –	1× Rh1	2.450
	2× Rh2	2.589
	1× Ga3	2.649
	1× Ga1	2.938
	1× Ga2	2.938
	1× Ga7	3.121
	1× Ga8	3.121
	1× Ga3	3.226
	1× Ga3	3.325
	1× Ga4	3.338
	2× Ga6	3.620
	1× Ga4	3.707
	1× Ga5	3.751
Ga6 –	1× Rh2	2.450
	2× Rh1	2.589
	1× Ga4	2.649
	1× Ga7	2.938
	1× Ga8	2.938
Ga7 –	2× Rh2	2.444
	2× Ga3	2.869
	2× Ga6	2.938

$P\bar{1}$_2		
Atoms		*d* /Å
Rh1 –	1× Ga3	2.430
	1× Ga1	2.444
	1× Ga2	2.444
	1× Ga5	2.450
	1× Ga4	2.555
	1× Ga6	2.589
	1× Rh1	2.883
Rh2–	1× Ga3	2.430
	1× Ga1	2.444
	1× Ga2	2.444
	1× Ga5	2.450
	1× Ga4	2.555
	1× Ga6	2.589
	1× Rh1	2.883
Ga1–	2× Rh1	2.444
	2× Ga5	2.938
	2× Ga4	2.959
	2× Ga3	2.972
Ga2–	2× Rh1	2.444
	2× Ga5	2.938
	2× Ga4	2.959
	2× Ga3	2.972
Ga3 –	1× Rh1	2.430
	1× Ga5	2.533
	2× Rh2	2.555
	1× Ga7	2.959
	1× Ga8	2.959
	1× Ga1	2.972
	1× Ga2	2.972
Ga4 –	1× Rh2	2.430
	1× Ga6	2.533
	2× Rh1	2.555
	1× Ga1	2.959
	1× Ga2	2.959
	1× Ga7	2.972
	1× Ga8	2.972
Ga5 –	1× Rh1	2.450
	1× Ga3	2.533
	2× Rh2	2.589
	1× Ga1	2.938
	1× Ga2	2.938
Ga6 –	1× Rh2	2.450
	1× Ga4	2.533
	2× Rh1	2.589
	1× Ga7	2.938
	1× Ga8	2.938
Ga7 –	2× Rh2	2.444
	2× Ga6	2.938

$P4_2nm$		
Atoms		*d* /Å
Rh –	1× Ga2	2.429
	2× Ga1	2.444
	1× Ga3	2.450
	2× Ga2	2.548
	2× Ga3	2.589
	1× Rh	2.883
Ga1–	2× Rh	2.444
	2× Ga2	2.868
	2× Ga3	2.938
Ga2–	1× Rh	2.429
	2× Rh	2.548
	2× Ga1	2.868
	1× Ga2	2.917
Ga3–	1× Ga3	2.350
	1× Rh	2.450
	2× Rh	2.589
	2× Ga1	2.938

$P4_22_12$		
Atoms		*d* /Å
Rh–	2× Ga2	2.389
	2× Ga1	2.444
	2× Ga2	3.533
	2× Ga2	2.554
	1× Rh	2.883
Ga1–	2× Rh	2.444
	2× Ga2	2.803
	2× Ga2	2.939
Ga2–	1× Rh	2.391
	1× Rh	2.535
	1× Rh	2.556
	1× Ga2	2.650
	1× Ga1	2.804
	1× Ga1	2.941

Ga8–	2× Rh2	2.444		2× Ga3	2.959
	2× Ga3	2.869		2× Ga4	2.972
	2× Ga6	2.938			
			Ga8 –	2× Rh2	2.444
				2× Ga6	2.938
				2× Ga3	2.959
				2× Ga4	2.972

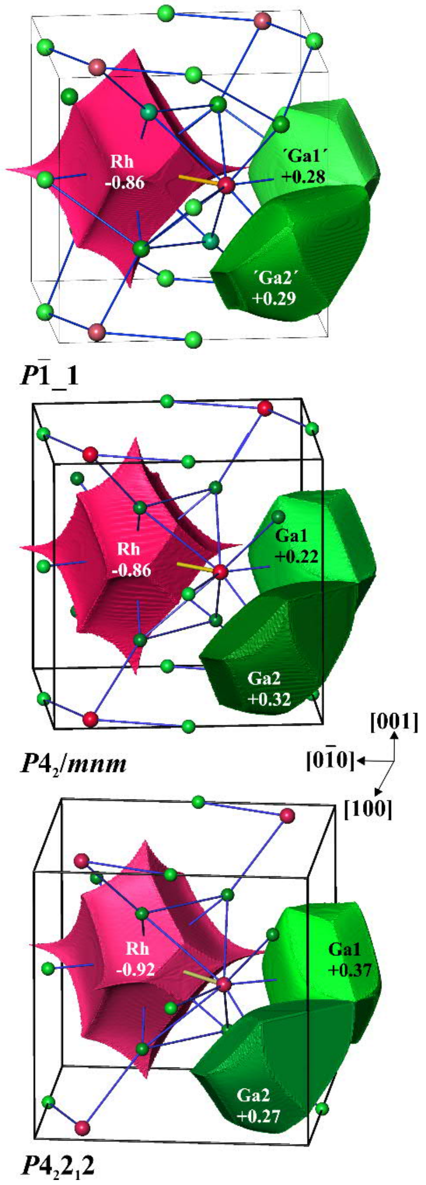

Figure S3. QTAIM atoms' basins and populations in the ordered models of *ht*-Ga_3Rh in comparison with the $IrIn_3$-type ($P4_2/mnm$) ones.

Quantitative analysis of the surfaces for the QTAIM Rh atoms: with 1.79 Å^2 or 3.6% of the total surface (49.84 Å^2) in the $P4_2nm$ model and 1.83 Å^2 or 3.6% of the total surface (51.36Å^2) in the $P\bar{1}$_2 model, the common Rh–Rh surfaces are clearly smaller than 2.43 Å^2 or 5.2% of the total surface (46.96 Å^2) in the $P4_2/mnm$ model.

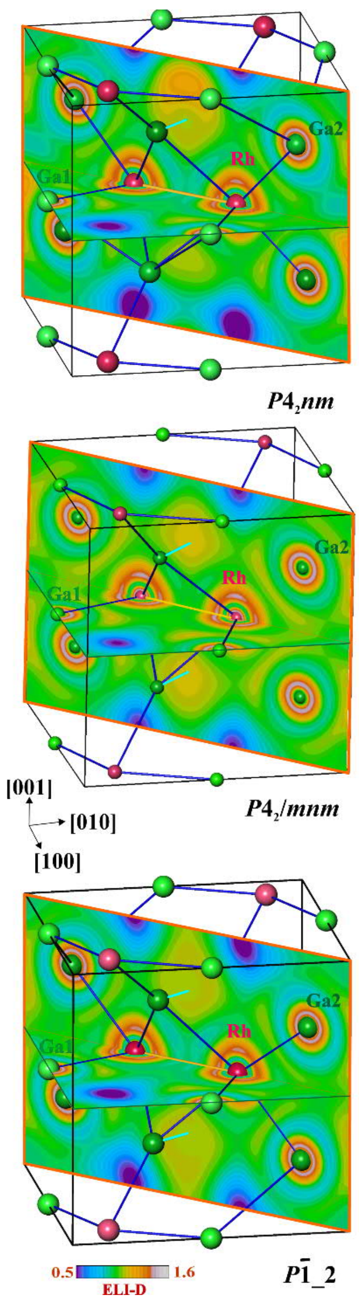

Figure S4. ELI-D distribution in the (001) and (110) planes in $P4_2/mnm$, $P4_2nm$ and $P\bar{1}\_2$ models of the crystal structure of *ht*-Ga_3Rh.

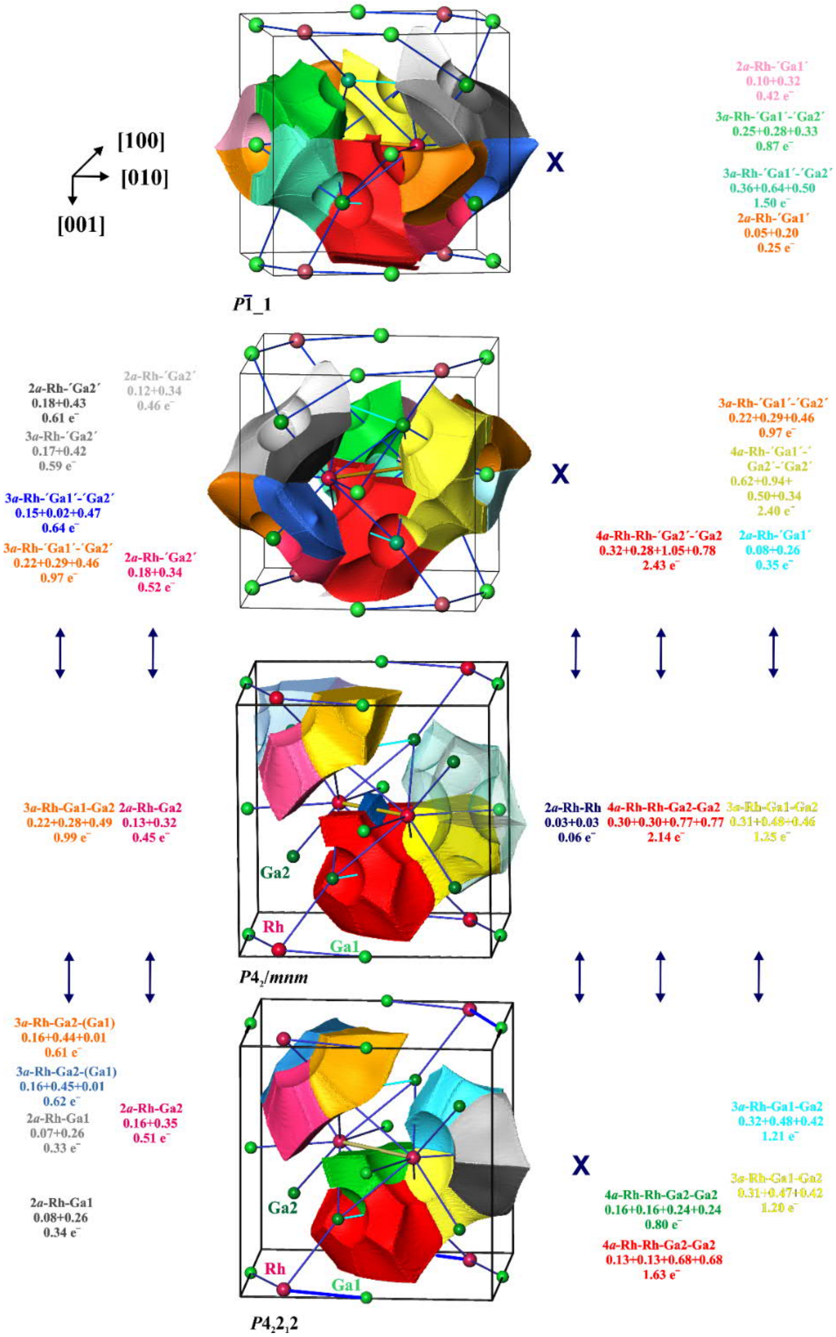

Figure S5. Bond basins, their atomicity and electron populations $P\bar{1}$_1 (top), $P4_2/mnm$ (middle) and in $P4_22_12$ (bottom) models of the crystal structure of *ht*-Ga_3Rh. The labels ´Ga1´, ´Ga2´ (bottom panel) denote the positions derived from initial Ga1, Ga2 (middle panel) due to the symmetry reduction. Dark-blue X means absence of the Rh–Rh bond basin. For the distortion modes cf. Figure S6. For the color-coding cf. Figure 7.

Analysis of the bond basin shapes gives the number of atoms whose core regions have a common surface with the basin of interest.

Due to the lower symmetry, the number of nine different bond types in the $P\bar{1}$_2 model is larger than in the $P4_2/mnm$ one (Figure 7, bottom). All heteroatomic bonds, known from the $P4_2/mnm$ model, are present in several variations also in the in the $P\bar{1}$_2 model. Here also, rhodium participates in all heteroatomic bonds as a minor partner. The characteristic difference to the $P4_2/mnm$ model, is the absence of the dedicated bond basin between the Rh atoms. It is only included in the 4*a*-Rh–Rh–Ga2–Ga2 bond basin (red basin in the bottom panels of Figure 7), but here the Ga2 contributions are the majority ones (72% of the bond population). This means that these two Ga2–Ga2 contacts within the TCRP should be included into the conceptual electron counting (2-electron bonds). That gives 19 bonds, which is not realizable with 36 available valence electrons. But it can be realized, if the one topologically absent Rh–Rh bond would be excluded (18 bonds). This agrees well with the ELI-D picture of the bonding (Figure 7, bottom panels).

Another way to describe the split in the difference electron density leads to a model with the space group $P4_2nm$ and trapezium-like shape of the middle quadrilateral plane of the TCRP (Figure 5, top, cf. also Experimental for atomic coordinates). Here, the number of different bond types – ten – is also larger than in the $P4_2/mnm$ one (Figure 7, top). The $P4_2nm$ model is similar to the $P\bar{1}$_2 one with respect to (i) the presence in several variations of all heteroatomic bond basins, known from the $P4_2/mnm$ model and (ii) the rhodium participation in all heteroatomic bonds as a minority partner. A dedicated bond basin between the Rh atoms is here also absent. In the bottom region of the unit cell, the original 4*a*-Rh–Rh–Ga2–Ga2 bond ($P4_2/mnm$ model, red basin in the middle panel of Figure 7) splits topologically into two 4-atomic parts (light red and

green basins in the top panel of Figure 7) with one of them (green basin) having more than 90% of the Ga22 contribution. On the conceptual level, the shorter Ga22–Ga22 contact and the Rh–Rh one in the TCRP should be included into electron counting. That gives 18 bonds, which can be realized with 36 available valence electrons, i.e. it agrees well with the ELI-D picture of the bonding (Figure 7, top panel). Similar results are obtained for the models $P\bar{1}$_2 and $P4_22_12$ (Figure S10).

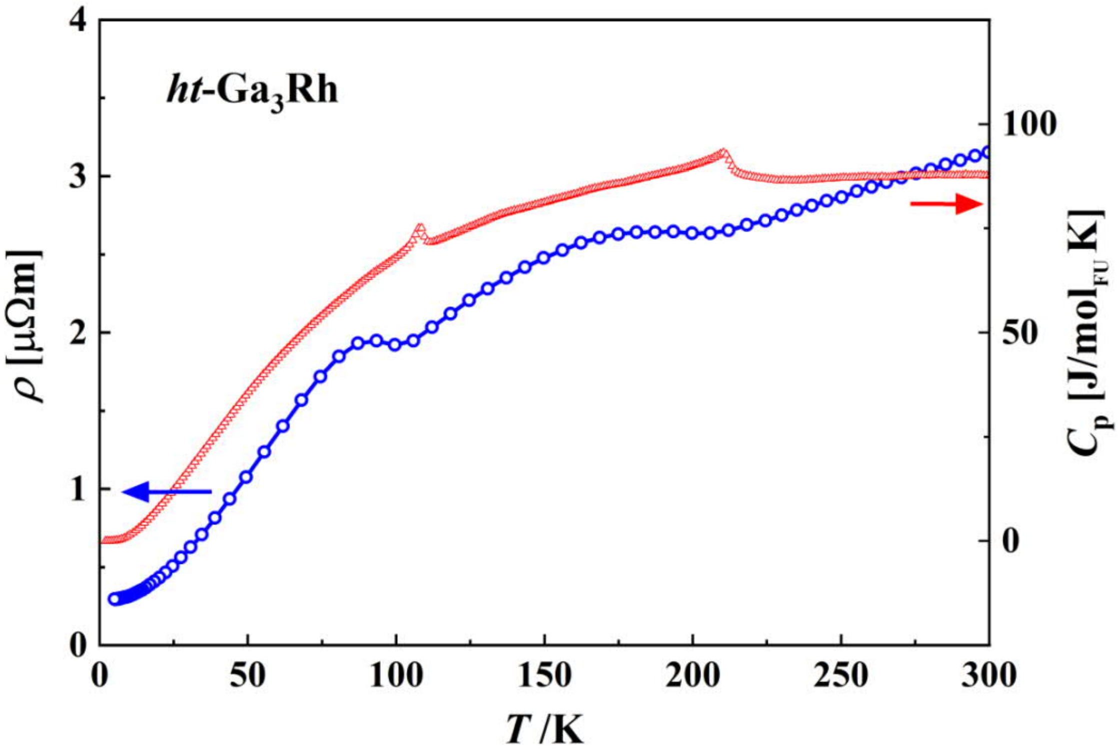

Figure S6. Specific heat C_P *versus* temperature for *ht*-Ga_3Rh in comparison with the temperature dependence of the resistivity revealing low-temperature transformations.
